# Predicting MHC class I epitopes in large datasets

**DOI:** 10.1186/1471-2105-11-90

**Published:** 2010-02-17

**Authors:** Kirsten Roomp, Iris Antes, Thomas Lengauer

**Affiliations:** 1 Department of Computational Biology and Applied Algorithmics, Max Planck Institute for Informatics, 66123 Saarbruecken, Germany; 2 Center for Integrated Protein Science Munich (CIPSM) and Department of Life Sciences, Technical University of Munich, 85354 Freising-Weihenstephan, Germany

## Abstract

**Background:**

Experimental screening of large sets of peptides with respect to their MHC binding capabilities is still very demanding due to the large number of possible peptide sequences and the extensive polymorphism of the MHC proteins. Therefore, there is significant interest in the development of computational methods for predicting the binding capability of peptides to MHC molecules, as a first step towards selecting peptides for actual screening.

**Results:**

We have examined the performance of four diverse MHC Class I prediction methods on comparatively large HLA-A and HLA-B allele peptide binding datasets extracted from the Immune Epitope Database and Analysis resource (IEDB). The chosen methods span a representative cross-section of available methodology for MHC binding predictions. Until the development of IEDB, such an analysis was not possible, as the available peptide sequence datasets were small and spread out over many separate efforts. We tested three datasets which differ in the IC_50 _cutoff criteria used to select the binders and non-binders. The best performance was achieved when predictions were performed on the dataset consisting only of strong binders (IC_50 _less than 10 nM) and clear non-binders (IC_50 _greater than 10,000 nM). In addition, robustness of the predictions was only achieved for alleles that were represented with a sufficiently large (greater than 200), balanced set of binders and non-binders.

**Conclusions:**

All four methods show good to excellent performance on the comprehensive datasets, with the artificial neural networks based method outperforming the other methods. However, all methods show pronounced difficulties in correctly categorizing intermediate binders.

## Background

A precise understanding of host immune responses is crucial for basic immunological studies as well as for designing effective disease prevention strategies. Epitope-based analysis methods are effective approaches at assessing immune response, allowing for the quantification of the interaction between a host and pathogen, of vaccine effectiveness or other prevention strategies.

As part of the adaptive immune response, antigens are recognized by two different types of receptor molecules: immunoglobulins which act as antigen receptors on B cells and antigen-specific T-cell receptors (TCRs) [1,2]. The latter receptor molecules recognize antigens which are displayed on the surface of cells. These antigens are peptide fragments derived from intracellular pathogens such as viruses or bacteria, or alternatively pathogens which have been endocytosed by the cells. The cytosolic degradation of pathogen proteins is carried out by a large, multicatalytic protease complex, the proteasome. Subsequently, the protein fragments are transported into the endoplasmic reticulum via the transporters associated with antigen processing (TAP), prior to being loaded onto the major histocompatibility complex (MHC) molecules, which are specialized host-cell glycoproteins that form a complex with the peptidic fragments. These fragments are then translocated to the cell surface as part of the MHC-peptide complex for TCR recognition. Peptides which trigger an immune response by this process are called T-cell epitopes. An alternative processing pathway is provided by the signal peptidase which bypasses the proteosome and TAP transport. The signal peptidase cleaves signal peptides from proteins entering the endoplasmic reticulum, which are then bound to MHC class I molecules. Particularly HLA-A*02 molecules, which prefer hydrophobic sequences, acquire peptides in this manner [3].

MHC class I molecules deliver peptides from the cytosol and are recognized by CD8^+ ^T cells. The binding of antigenic peptides from pathogens to MHC class I molecules is one of the crucial steps in the immunological response against an infectious pathogen [2]. While not all peptides that bind MHC molecules become epitopes, all T-cell epitopes need to bind to MHC molecules. Therefore, deciphering why certain peptides become epitopes and others do not is central to the development of a precise understanding of host immune responses.

The Immune Epitope Database and Analysis Resource (IEDB) [4,5] is a central data repository and service, containing MHC binding data relating to B cell and T cell epitopes from infectious pathogens, experimental pathogens and self-antigens (autoantigens). In most cases, T cell epitopes are defined as peptides that are not only presented to T-cell receptors on the cell surface by specific MHC molecules, but that also trigger an immune response. IEDB encompasses patent data from biotechnological and pharmaceutical companies, as well as direct submissions from research programs and partners. Within the database, epitopes are linked with objective and quantifiable measurements with regard to their binding affinity to specific, well defined immune system receptors.

IEDB is not the first database to store such information, as there are a number of databases which include similar information. However, although most of the components of IEDB can be found in other resources, none contains them all. For example, SYFPEITHI [6] contains carefully mapped epitopes or naturally processed peptides, but unlikely IEDB, does not annotate the context in which they are immunogenic. The Los Alamos HIV Molecular Immunology Database [7], focuses on a restricted dataset. FIMM [8], is of modest size and solely focuses on cellular immunology and MHCPEP [9], while still widely used, has not been updated since 1998. While MHCBN [10] and AntiJen [11,12] contain peptide entries that are not contained in IEDB, IEDB has more entries than any other existing database in this field.

While IEDB is the first epitope database of significant size, the experimental screening of large sets of peptides with respect to their MHC binding capabilities is still very demanding due to the large number of possible peptide sequences and the extensive polymorphism of the MHC proteins. Therefore, there is significant interest in the development of computational methods for predicting the binding capability of peptides to MHC molecules, as a first step towards selecting peptides for actual screening.

Sequence- and structure-based methods, as well as combinations thereof, have been developed and were used for both classification and regression. Classification models aim to distinguish binding from non-binding peptides, whereas regression methods attempt to predict the binding affinity of peptides to MHC molecules. As the quantity of publicly available binding data has been limited until recently, most methods focus on classification. A review of previous methods can be found in Tong *et al*. [13].

Sequence-based methods are computationally more efficient than structure-based methods. However, they are hampered by the need for sufficient experimental data and therefore only achieve high performance on already intensively investigated MHC alleles. Additionally, sequence-based methods do not provide a structural interpretation of their results, which is of importance for designing peptidic vaccines and drug-like molecules. Structure-based methods have the advantage of being independent of the amount of available experimental binding data, but are computationally intensive and therefore not suited for the screening of large datasets.

A recent approach [14] performs a combined structure-sequence-based prediction by incorporating structural information obtained from molecular modeling into a sequence-based prediction model. This method therefore not only allows for the fast prediction of MHC class I binders, but also for the efficient construction of docked peptide conformations. This approach is the only prediction method available today, which also allows for the construction of such conformations. We have evaluated this approach for MHC class I alleles of the human leukocyte antigen (HLA) genes A and B for which extensive datasets were available in IEDB and compared it to two sequence-based prediction methods from the literature. These two sequence-based prediction methods are the same as examined in Antes *et al*. [14] and were chosen here as well for comparison reasons. In addition, we have evaluated the prediction server *NetMHC *which has shown to be among the best predictors in recent comparison tests [15].

A major focus of this study is on testing the dependency in performance of well established methods on the use of different training and testing datasets. The four methods we have chosen span a representative cross section of available methodology for MHC-peptide binding predictions, from simple binary (*SVMHC*) to rather sophisticated encoding (*DynaPred*^*POS*^). The chosen methods include advanced learning strategies such as support vector machines (SVM) (*DynaPred*^*POS *^and *SVMHC*) and artificial neural networks (ANN) (*NetMHC*), as well as the more straightforward quantitative matrix based prediction (*YKW*).

Lin et al [15] performed a comparative evaluation of thirty prediction servers developed by 19 groups using an independent dataset. Each server was accessed via the Internet and the predictions were recorded, normalized, and compared. Peters et al [16] performed an extensive analysis of predictors, in which they trained and tested their in-house methods, but did not reimplement any of the external methods used. Instead, web interfaces were used for the external methods. Zhang et al [17] evaluated five prediction methods using public web interfaces with the default parameters of the methods in question. Three of these methods were in-house and two were external. The authors discarded all peptides used for training their own methods for subsequent testing, but there was some concern that there was some overlap between evaluation and training data sets for one of the external methods. They also trained their own predictors on a dataset of binders and non-binders for a wide variety of alleles, testing on a second set of binders and non-binders which were released at a later point in time. The goal of this analysis was to examine the performance of these predictors on alleles for which little or no data was available (which were described as pan-specific predictors). The alleles for which such pan-specific analyses were performed were not identified and only limited information on the methods performance was available.

Work by other groups which preceded the study by Zhang et al [17], included a support vector machine based approach [18], which was trained and tested on a relatively small datasets, where different predictive models are estimated for different alleles, using training data from 'similar' alleles. The notion of allele similarity is defined by the user therefore requiring human intervention which is not a systematic procedure. A binding energy model [19], which was trained and tested on very small datasets, was used to make pan-specific predictions for only two alleles. A further study [20], which utilized hidden Markov models and artificial neural networks as predictive engines, was again trained on relatively small datasets. The system was used to identify so-called promiscuous peptides, which bind well to a number of diverse alleles.

In this study, we reimplemented the external methods *YKW *and *SVMHC *and trained and tested them along side our in-house method *DynaPred*^*POS *^on a wide variety of datasets. This allows for a more objective comparison of the performance of these methods. We also tested *NetMHC *in all the tests where training was not necessary; this server is only available via a web interface and thus could not be reimplemented for this study. In contrast to the previous work described, we perform a detailed analysis of the performance of predictors trained on one allele and their ability to accurately predict other alleles.

## Methods

### Datasets with Complete Peptides

In this section we describe the datasets we use that contain data incorporating full peptides, i.e. information on all nine residues. IEDB was mined for allele/peptide data on May 16th, 2007. Only alleles with a significant number of 9-mer binding and non-binding peptides (the total number being greater than 200) where included in the analysis (Table 1). The data was imported into a local relational database to allow for efficient analysis.

**Table 1:** Prediction accuracies for the full dataset

	Allele Name	Binders	Non-Binders	Total	DynaPredPOS	NetMHC	SVMHC	YKW
		*(n)*	*(n)*	*(n)*	*AUC*	*AUC*	*AUC*	*AUC*
1	A*0101	163	1316	1479	0.93	0.98	0.95	0.94
2	A*0201	1544	1929	3473	0.93	0.96	0.92	0.91
3	A*0202	723	697	1420	0.88	0.93	0.85	0.85
4	A*0203	732	685	1417	0.88	0.95	0.86	0.84
5	A*0206	633	782	1415	0.88	0.95	0.87	0.86
6	A*0301	637	1618	2255	0.89	0.96	0.88	0.80
7	A*1101	816	1279	2095	0.92	0.96	0.90	0.91
8	A*2402	202	464	666	0.80	0.85	0.78	0.81
9	A*2601	69	885	954	0.84	0.93	0.83	0.84
10	A*3101	510	1480	1990	0.89	0.95	0.89	0.88
11	A*3301	203	994	1197	0.88	0.96	0.89	0.88
12	A*6801	578	620	1198	0.85	0.92	0.82	0.81
13	A*6802	439	980	1419	0.84	0.93	0.85	0.83
14	B*0702	238	1110	1348	0.94	0.98	0.94	0.93
15	B*0801	23	687	710	0.82	0.99	0.78	0.79
16	B*1501	182	836	1018	0.86	0.97	0.89	0.90
17	B*2705	81	917	998	0.93	0.97	0.90	0.94
18	B*3501	273	578	851	0.83	0.93	0.84	0.85
19	B*4001	94	1112	1206	0.90	0.97	0.93	0.91
20	B*4402	76	136	212	0.77	0.84	0.75	0.76
21	B*4403	71	142	213	0.68	0.81	0.65	0.70
22	B*5101	108	249	357	0.82	0.93	0.80	0.81
23	B*5301	127	228	355	0.86	0.95	0.85	0.87
24	B*5801	78	893	971	0.90	0.99	0.93	0.93
	**Average AUC**				**0.86**	**0.94**	**0.86**	**0.86**

Alleles included in the study with the number of binders and non-binders available; all available binders and non-binders were included in the analysis irrespective of whether quantitative laboratory test data was available or not (Dataset F). Only unique peptide sequences were included in the counts; all peptides with more than one entry for a particular allele in IEDB were counted once only. The overall performance of the four prediction models on different alleles is shown; AUC = area under the curve (ROC analysis). The average AUC for each method is included at the bottom of each column.

Three datasets were generated for each allele: all peptides available in IEDB (the full dataset or Dataset F), all peptides with an available quantitative laboratory test result (IC_50 _or in rare cases EC_50_), but including only those with a binding affinity between 50 nM and 1000 nM, i.e. including only weak binders and non-binders (the intermediate dataset or Dataset I) and all peptides with an IC_50_, but excluding those with a binding affinity between 10 nM and 10,000 nM, i.e. including only very strong binders and very clear non-binders (the strong dataset or Dataset S). Alleles with less than 200 peptides in total (binders and non-binders) were excluded from the analysis in all datasets. IC_50 _measures the half maximal (50%) inhibitory concentration (IC) of a radioactive isotope labeled standard peptide to MHC molecules, whereas EC_50 _measures the half maximal effective concentration (EC) of such a reference peptide [4,5]. For Dataset F, in cases where a peptide with a particular sequence had more than one entry in IEDB for a particular allele (for example the peptide was tested with same allele by two different laboratories, resulting in two separate IEDB entries), this peptide was included only once. If no binding constant was available, the peptide was also only included once in Dataset F. If a peptide was described as a binder by one laboratory and a non-binder by another laboratory, it was included both as a binder and non-binder in Dataset F. For Datasets I and S, in cases where peptide-allele complexes had duplicate entries in IEDB and the binding affinities differed, resulting in at least one entry with a binding affinity which fell within the ranges used in Datasets I and S, this peptide was included in the respective dataset. If, for a particular allele, there was more than one binding affinity measurement made that fell into one of the ranges used in the analysis, an average binding affinity was calculated and used for that peptide in that particular range. Any peptides annotated in IEDB as binders with IC_50 _values greater than 500 nM, and peptides annotated as non-binders with IC_50 _values less than 500 nM were discarded. We have made the three datasets available: http://www.mpi-inf.mpg.de/~roomp/benchmarks/list.htm.

We also tested an independent dataset recently published by Lin et al [15], derived from the tumor antigen survivin and the cytomegalovirus internal matrix protein pp65. This data was not used for training any of the four prediction methods in this study and therefore serves as an independent test set.

### Prediction Methods

Four prediction methods were evaluated with respect to their ability to correctly classify binders and non-binders in the datasets described above. As already described in the introduction, the chosen methods span a representative cross-section of available methodology for MHC binding predictions.

The first prediction method used (*DynaPred*^*POS*^) was developed in our laboratory [14]. The general strategy followed to generate this prediction model, involves as a first step molecular dynamics simulations from which energetic information for all 20 amino acids in each of the nine binding pockets of the binding groove of HLA-A*0201 was extracted. The algorithm is based on the assumption that the total binding affinity of a peptide can be approximated by the sum of the binding affinities of its individual amino acids, neglecting the effect of interactions between neighboring residues. Therefore, each amino acid was simulated individually in each binding pocket; initial conformations were constructed from available crystal structures and, in order to stabilize the peptide conformations, the single so-called pivot amino acid was extended by a glycine residue on both sides (for terminating residues on the non-terminating side only) resulting in pseudo-dimers or -trimers which were used in the simulations. For amino acids with no available experimental structures, existing residues were mutated to the corresponding amino acid using the program SCWRL3 [21]. Subsequently, a binding-free-energy-based scoring matrix (BFESM) was constructed which included important energy terms reflecting the binding properties of the amino acids derived from the simulations. Each entry in the matrix represented one feature (energy term) of a particular amino acid in a particular binding pocket.

The BFESM is used to generate a feature vector for each given peptide in the training dataset; all vectors together produce a feature matrix for model generation and prediction. A local feature matrix is constructed from the BFESM which uses all residue and binding pocket positional information from the scoring matrix. This matrix provides a basis for logistic regression and SVM training [22] of the final model (*DynaPred*^*POS*^).

One feature unique to *DynaPred*^*POS *^is the ability to construct bound peptide conformations for all predicted sequences. The bound conformations are generated by connecting the saved residue conformations for the simulation runs and performing a short energy minimization. In a detailed analysis [14], the constructed peptide structures were refined within seconds to structures with an average backbone RMSD of 1.53 Å from the corresponding experimental structure.

Additionally, we evaluated two sequence-based prediction methods from the literature. The first method is *SVMHC *from Dönnes *et al*. [23], which is based on SVMs and was implemented using the software package SVM-LIGHT [24]. For this method, SVM kernels and trade offs were optimized by systematic variation of the parameters and evaluation of prediction performance was made using Matthews Correlation coefficients [25], which were used as the main measure of performance for parameter optimization. The second sequence-based prediction method we evaluated is *YKW *from Yu *et al*. [26], which is based on data-derived matrices. The matrix is generated using logarithmized propensities for occurrence in binding vs. nonbinding peptides of amino acids at specific positions within the peptide training set to generate an initial matrix. The final matrix was derived by a position dependent weighting of the initial matrix which was derived by an analysis of binding data. The SVMHC and YKW methods were re-implemented for this study using the methodology reported in the original publications.

The fourth and final method we evaluated is an artificial neural network based approach [27,28], which was developed using ANN which are capable of performing sensitive, quantitative predictions. Such quantitative ANN were shown to be superior to conventional classification ANNs which have been trained to predict binding versus non-binding peptides. *NetMHC *has recently been shown to be among the best predictors in an extensive comparison of prediction servers whose performance was evaluated with 176 peptides derived from the tumor antigen survivin and the cytomegalovirus internal matrix protein pp65 [15]. *NetMHC *is available via http://www.cbs.dtu.dk/services/NetMHC/. *NetMHC *could not be trained for this study as it was only accessible via with web interface, and was therefore used for testing purposes only. Also, it is probable, that at least some of the data used to train the *NetMHC *server was the same data which was retrieved from IEDB for this study.

For Datasets F, I and S, training and testing of the prediction models for *SVMHC, YKW*, and *DynaPred*^*POS *^was performed for each HLA-A and HLA-B allele separately. In the case of *DynaPred*^*POS*^, the same BFESM generated from the molecular simulations on A*0201 was used to generate each new feature matrix for each allele separately. For *NetMHC*, the peptide sequences from Dataset F, I and S were submitted to the prediction server and the prediction results were recorded.

The accuracy of the methods was assessed by generating areas under the curve (AUC, see ROC analysis [29]), which is a widely used non-parametric performance measure. ROC analysis tests the ability of models to separate binders from non-binders without the need of selecting a threshold. The values AUC ≥ 0.90 indicate excellent, 0.90 > AUC ≥ 0.80 good, 0.80 > AUC ≥ 0.70 marginal and 0.70 > AUC poor predictions [30].

We used 10-fold cross validation to assess the accuracy of the predictions.

### Robustness

In order to determine how dependent the reproducibility of the results of the prediction methods *YKW*, *SVMHC *and *DynaPred*^*POS *^are on the size of the available allele datasets (a phenomenon that we call robustness), we tested the methods' performance with randomly selected balanced datasets of different sizes, selected from all peptides available in IEDB for a particular allele (Dataset F). *NetMHC *was not included in this analysis, because we were unable to retrain the statistical model.

The alleles examined were A*0201, A*3101 and B*0702. The training was performed on each allele separately, followed by testing using 10-fold cross validation. The smallest balanced dataset for each allele consisted of 50 randomly selected binders and 50 randomly selected non-binders and the size of the largest dataset depended on the overall number of binders or non-binders available for the allele. All prediction methods were run on four randomly selected balanced datasets in each size category for each allele.

### Generalizability

By this test, we assess the ability of the statistical models (*YKW*, *SVMHC *and *DynaPred*^*POS*^), trained for the allele A*0201, to generalize to other alleles. *NetMHC *was again not included in this analysis, because we were unable to retrain the statistical model. Training was performed on Dataset F of A*0201, followed by testing on Datasets F of all other alleles. This generalization ability is essential for epitope prediction models as there are many alleles with insufficient data for training an allele-specific model.

## Results and Discussion

### Datasets with Complete Peptides

In this section we examine the dependency of the performance of the four prediction methods on the selection criteria of the used training dataset.

If all available peptides for an allele are used for the prediction (Table 1, Dataset F), *NetMHC *performs particularly well, achieving the highest AUC for all 24 alleles examined. All four methods had a predictive performance of good or excellent for 20 or more of the 24 alleles. *NetMHC *significantly outperforms the three other methods (Wilcoxon Rank Sum Test, P-value < 0.001) and no statistically significant difference between the other three methods could be detected. Therefore, the ranking of the methods can be described as *NetMHC *> (*DynaPred*^*POS*^, *SVMHC*, *YKW*).

The results are dependent on the size of the datasets: for good results (AUC greater than 0.85), an allele's dataset generally has to contain more than 100 binders and more than 100 non-binders (preferably more than 200 binders and more than 200 non-binders). Also, datasets for which the number of binders and non-binders are relatively balanced produced larger AUCs (i.e. better performance). Unbalanced datasets in IEDB generally have a substantially lower number of binders than non-binders. For *YKW*, *SVMHC *and *DynaPred*^*POS *^better results were achieved for HLA-A than HLA-B. This probably is due to the lower number of epitopes which are available for HLA-B in IEDB and, in the case of *DynaPred*^*POS*^, due to the fact that the BFESM was generated using HLA-A*0201 simulation results. *NetMHC *however achieved comparable results for both HLA-A than HLA-B.

Intermediate binders (Table 2, Dataset I) were difficult to classify. *NetMHC *had the best performance for 10 out of 11 alleles. However, all methods showed at best marginal prediction performance (the largest achieved AUC was 0.79) and in most cases the predictions were poor.

**Table 2:** Prediction accuracies for the dataset containing only weak binders and non-binders

	Allele Name	Binders	Non-Binders	Total	DynaPredPOS	NetMHC	SVMHC	YKW
		*(n)*	*(n)*	*(n)*	*AUC*	*AUC*	*AUC*	*AUC*
1	A*0201	616	135	751	0.67	0.77	0.65	0.66
2	A*0202	286	87	373	0.53	0.70	0.41	0.54
3	A*0203	261	126	387	0.58	0.79	0.58	0.60
4	A*0206	264	74	338	0.56	0.73	0.57	0.57
5	A*0301	335	106	441	0.58	0.70	0.60	0.59
6	A*1101	374	91	465	0.56	0.69	0.62	0.63
7	A*3101	278	103	381	0.42	0.69	0.48	0.56
8	A*3301	129	72	201	0.62	0.52	0.39	0.63
9	A*6801	273	96	369	0.54	0.68	0.44	0.57
10	A*6802	227	123	350	0.52	0.74	0.50	0.50
11	B*1501	169	33	202	0.59	0.79	0.49	0.59
	**Average AUC**				**0.56**	**0.71**	**0.52**	**0.59**

Results from Dataset I, in which only weak binders (50 nM to 500 nM binding affinity) and non-binders (500 nM to 1000 nM binding affinity) were included. Alleles included in Dataset F, which had fewer than 200 binders and non-binders in total in Dataset I, were no longer included in the analysis. The average AUC for each method is included at the bottom of each column.

**Figure 1:**
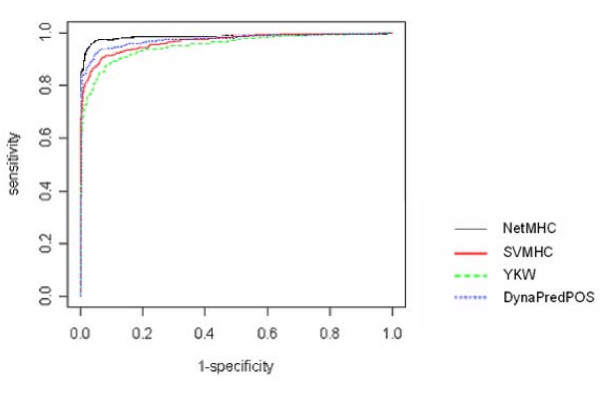
**Overall performance evaluation**. ROC plot for the overall performance evaluation of *SVMHC*, *YKW*, *DynaPred*^*POS *^with models that are trained and tested on Dataset S pertaining to allele A*0201. *NetMHC *was only available online and therefore could not be trained; the results shown result from testing with Dataset S.

Restricting the datasets to peptides which were either very strong binders or clear non-binders substantially improved the results in most cases (Table 3, Dataset S); with thirteen of fourteen alleles the best method, *NetMHC*, achieved AUC equal to or greater than 0.99. With the exception of allele A*2402, all methods had an excellent predictive performance (AUC greater than 0.90). Despite a substantially lower number of data points in Dataset S, a higher accuracy was found for the best method for all alleles when compared with the Datasets F. A typical ROC plot comparing the performance of the four prediction methods for Dataset S is shown in Figure 1.

**Table 3:** Prediction accuracies for the dataset containing only strong binders and clear non-binders

	Allele Name	Binders	Non-Binders	Total	DynaPredPOS	NetMHC	SVMHC	YKW
		*(n)*	*(n)*	*(n)*	*AUC*	*AUC*	*AUC*	*AUC*
1	A*0101	34	284	318	0.96	1.00	0.96	0.97
2	A*0201	549	503	1052	0.97	0.99	0.97	0.95
3	A*0202	290	267	557	0.98	1.00	0.97	0.97
4	A*0203	273	255	528	0.97	0.99	0.96	0.96
5	A*0206	216	371	587	0.97	0.99	0.96	0.95
6	A*0301	123	332	455	0.93	1.00	0.94	0.95
7	A*1101	228	269	497	0.95	1.00	0.94	0.97
8	A*2402	69	272	341	0.88	0.84	0.84	0.85
9	A*2601	15	256	271	0.97	1.00	0.94	0.97
10	A*3101	114	349	463	0.96	1.00	0.96	0.97
11	A*3301	36	620	656	0.95	1.00	0.94	0.93
12	A*6801	155	235	390	0.95	1.00	0.94	0.94
13	A*6802	95	440	535	0.96	1.00	0.97	0.94
14	B*0702	45	161	206	0.95	1.00	0.96	0.93
	**Average AUC**				**0.95**	**0.99**	**0.95**	**0.95**

Results from Dataset S, in which only strong binders (less than 10 nM binding affinity) and very clear non-binders (greater than 10,000 nM binding affinity) were included. Alleles included in Dataset F, which had fewer than 200 binders and non-binders in total in Dataset S, were no longer included in the analysis. The average AUC for each method is included at the bottom of each column.

Overall, the best performance was achieved in cases where Dataset S was used, the number of binders in the dataset was large (more than 200 binders and more than 200 non-binders), the dataset was relatively well balanced, the *NetMHC *method was used, and the allele was of the HLA-A*02 type.

For the independent dataset of 176 peptides (Table 4), while *NetMHC *was the best method for five of the seven alleles tested there was no significant difference in the performance of the methods. For all alleles, with the exception of A*1101, at least one method had excellent predictive performance (AUC greater than 0.90); generally at least two methods showed excellent predictive performance.

**Table 4:** Prediction accuracies for an independent dataset

	Allele	Binders	Non-Binders	Total	DynaPredPOS	NetMHC	SVMHC	YKW
		*(n)*	*(n)*	*(n)*	*AUC*	*AUC*	*AUC*	*AUC*
1	A*0201	33	143	176	0.92	0.94	0.82	0.93
2	A*0301	11	165	176	0.77	0.92	0.70	0.85
3	A*1101	17	159	176	0.84	0.89	0.72	0.83
4	A*2402	37	139	176	0.90	0.78	0.90	0.64
5	B*0702	9	167	176	0.86	0.98	0.72	0.68
6	B*0801	10	166	176	0.97	0.92	0.97	0.61
7	B*1501	14	162	176	0.71	0.92	0.71	0.80
	**Average AUC**				**0.85**	**0.90**	**0.79**	**0.76**

A set of 176 novel peptides, generated and tested by Lin et al [15], were used to test the prediction accuracy of the four methods in this study. The average AUC for each method is included at the bottom of each column.

### Robustness

**Figure 2:**
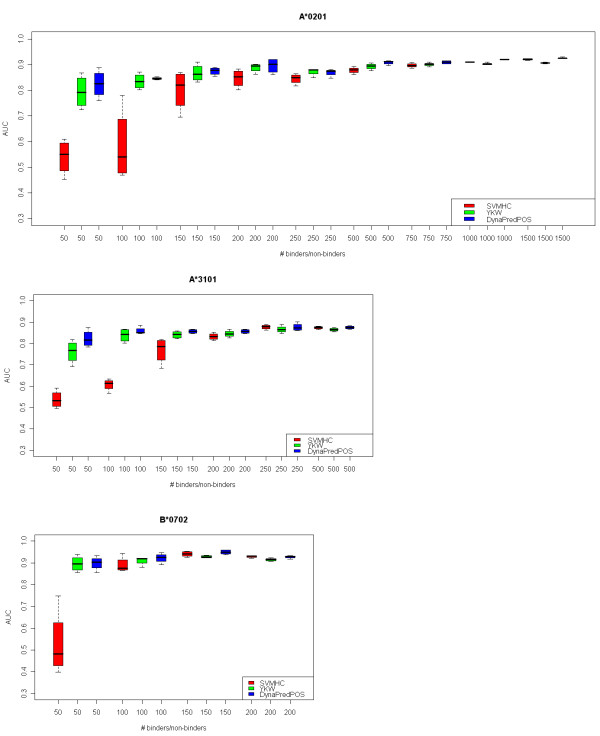
**Robustness analysis**. The reproducibility of the results of the prediction methods and their dependence on the size of the available dataset was examined in selected alleles. Box plots of randomly selected balanced sets of binders and non-binders from Dataset F for the alleles A*0201, A*3101, and B*0702 are shown. The smallest dataset for each allele consisted of 50 binders and 50 non-binders. The size of the largest dataset for each allele depends on the total number of binders or non-binders available for that particular allele. *NetMHC *was not included in this analysis as the predictor is only available online and could therefore not be trained by the authors.

In this test we examined the dependency of the quality of the obtained prediction models (*YKW*, *SVMHC *and *DynaPred*^*POS*^) on the size of the training sets used. *NetMHC *was not included in this analysis as the predictor is only available online and therefore could not be trained by the authors. We found that in most cases the AUCs stabilized at or close to their maximum level, when the size of the randomly selected balanced dataset consisted of more than 200 binders and 200 non-binders (Figure 2). This effect was observed with all three prediction methods and for all three alleles included in the study. *SVMHC *performance was less stable for small datasets, which might be due to its simple encoding method and is a significant drawback of this method.

### Generalizability

**Figure 3:**
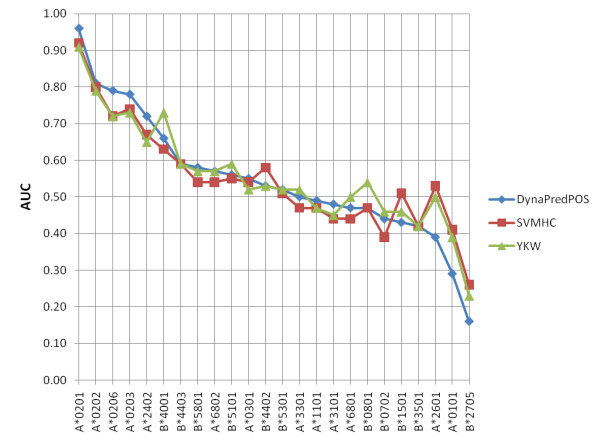
**Performance Comparison on Dataset F**. The performance of the three prediction models, trained on Dataset F of A*0201 and tested on Dataset F of all alleles (AUCs). *NetMHC *was not included in this analysis as the predictor is only available online and therefore could not be trained by the authors.

Last, we evaluated the generalizability of *YKW*, *SVMHC *and *DynaPred*^*POS *^on all HLA-A and HLA-B alleles for Datasets F. *NetMHC *was again not included in this test because the model could not be trained by the authors. In Figure 3 the AUCs for the models trained on the A*0201 dataset is given for different alleles. It can be seen that *DynaPred*^*POS *^outperforms the other models for alleles of the HLA-A*02 type, but for the other alleles the performance of the three methods is very similar. The prediction capabilities are good to marginal for some alleles implying that cross-allele prediction is feasible in some cases.

The MHC supertype classifications schemes generate clustered sets of molecules with largely overlapping peptide repertoires [31,32]. These classification schemes generally depend on features such as published motifs, binding data and the analysis of shared repertoires of binding peptides, etc. There has been interest in the development of pan-specific algorithms that can predict peptide binding to alleles for which limited or even no experimental data is available. This would, in contrast to the typical supertype classification scheme which depends on the availability to such data, allow for the prediction of binding in cases where no such data is available.

In recent work by Zhang et al. [17], their predictor was trained on a dataset of binders and non-binders for a wide variety of alleles, and tested on a second set of datasets which consisted of binders and non-binders which were released at later point in time. While the study claims to analyze performance on alleles for which no or only limited data is available, these alleles are never identified and only very limited information on the results is given. Also, the performance on alleles for which no data was available for training was poor. In contrast, we have performed a detailed analysis of the performance of predictors trained on one allele, and their ability to accurately predict other alleles.

There have been several papers defining supertypes using a number of different approaches [32,33]. Generally A*02 alleles and A*24 alleles are not clustered in the same supertype. Our generalizability work was however able to make reasonable predictions for A*2402 (the only allele in our study of this supertype), using a predictor trained on allele A*0201.

## Conclusions

Until the creation of IEDB, the available peptide sequence datasets were small and spread over many separate efforts. In addition the datasets consisted predominantly of binding sequences, so that most prediction models based on these data used random non-binding data for training purposes. Through the IEDB database a sufficiently large number of experimentally verified non-binders have become available for learning for the first time. Therefore, the prediction models evaluated here could not only be tested on a substantially larger number of binders, but in addition experimentally verified non-binders could be included in the training datasets and alleles that have previously not been analyzed due to insufficient dataset sizes could be included in the study.

As expected, Dataset S, which consisted only of peptides for which a quantitative laboratory result was available in IEDB and which were either strong binders or clear non-binders, performed better than Dataset F. As the binding affinity at which a binder becomes a non-binder has a threshold of 500 nM in IEDB, removing all peptides from the dataset which we described as so-called intermediate binders (50 nM to 1000 nM) improved the performance of the methods (results not shown), as did using a subset of data containing only the very strongest binders and clearest non-binders. Due to the error involved in experimental binding affinity analysis [34], we suggest using a cutoff of 500 nM may incorrectly categorize a weak binder as a non-binder or vice versa. Perhaps adding a category containing such intermediate binders, in addition to the already existing categories binder and non-binder, would be a useful addition to IEDB.

The excellent performance of *NetMHC*, on Dataset S in particular where it performs with an AUC of 1 for many alleles, may be in part due to the fact that this method could not be trained for the purposes of this study as *NetMHC *was only accessible via a web interface. It should also be noted that some of the data used to train *NetMHC *was probably identical to that extracted from IEDB for this study. This conjecture is also supported by the prediction results of the methods on the independent, novel dataset, which showed no statistically significant results between the methods (Table 4).

Peters et al. [16] performed an extensive analysis in which they trained and tested their own in-house methods, but external methods were not reimplemented by the authors. Instead, the available web interfaces for external methods were used with the default settings. In contrast to this, we reimplemented the external methods *YKW *and *SVMHC *to allow for both training and testing.

The analysis of the reproducibility of the results of the examined prediction models and their dependence on the size of the available dataset (robustness) showed that all methods require a sufficient number of data points for reproducible results (Figure 2). Overall, most alleles appear to require a minimum of 200 binders and 200 non-binders in Dataset F before the AUCs stabilize at or close to their maximum level. We suggest that performing the analysis on alleles with too few data points (which is still unfortunately the case with many HLA*B alleles) can lead to unreliable results.

The analysis of the methods' generalizability showed that the prediction capabilities are good to marginal for some alleles, implying that cross-allele prediction is feasible in some cases. In other cases, the AUCs were very low. Having trained with A*0201 and then tested for generalizability on HLA-A and HLA-B alleles in Dataset F, a possible reason for certain alleles to give rise to such low AUCs may be that a particular subset of binders that bind well to A*0201 may be very clear non-binders for the alleles in question. Conversely, clear non-binders for A*0201 may be binders for the other alleles leading to low AUCs.

In contrast to the former study [14], which included only binding sequences (as sufficient numbers of experimentally verified non-binding sequences were not available at that time) in testing generalizability, we observed a much improved level of cross-allele prediction with these newer datasets.

## Authors' contributions

KR contributed to the design of the study, computed and interpreted the results and drafted the manuscript. IA conceived the study, participated in its design and the interpretation of the results, and helped draft the manuscript. TL contributed to the design of the study and to the interpretation of results and helped draft the manuscript. All authors contributed to the writing of the manuscript and read and approved the final version.
